# Functional dissociation of hippocampal subregions corresponding to memory types and stages

**DOI:** 10.1186/s40101-020-00225-x

**Published:** 2020-07-02

**Authors:** Ji-Woo Seok, Chaejoon Cheong

**Affiliations:** 1 Department of Psychiatry, University of Nebraska Medical Center, Omaha, NE USA; 2 Department of Rehabilitation Counseling Psychology, Seoul Hanyoung University, Seoul, Republic of Korea; 3 Center for Research Equipment, Korea Basic Science Institute, 162 Yeongudanji-ro, Ochang, Cheongju, 28119 Chungbook Republic of Korea

**Keywords:** Hippocampus, Ultra-high field fMRI, CA 1–3, Subiculum, Implicit memory, Explicit memory, Encoding, Retrieval

## Abstract

**Background:**

The hippocampus reportedly plays a crucial role in memory. However, examining individual human hippocampal-subfield function remains challenging because of their small sizes and convoluted structures. Here, we identified hippocampal subregions involved in memory types (implicit and explicit memory) and stages (encoding and retrieval).

**Methods:**

We modified the serial reaction time task to examine four memory types, i.e. implicit encoding, explicit encoding, implicit retrieval, and explicit retrieval. During this task, 7-T functional magnetic resonance imaging was used to compare brain activity evoked by these memory types.

**Results:**

We found hippocampal activation according to all memory types and stages and identified that the hippocampus subserves both implicit and explicit memory processing. Moreover, we confirmed that cornu ammonis (CA) regions 1–3 were implicated in both memory encoding and retrieval, whereas the subiculum was implicated only in memory retrieval. We also found that CA 1–3 was activated more for explicit than implicit memory.

**Conclusions:**

These results elucidate human hippocampal-subfield functioning underlying memory and may support future investigations into hippocampal-subfield functioning in health and neurodegenerative disease.

## Background

Long-term human memory can be divided into two categories: explicit and implicit. Explicit memory is the conscious and intentional recall of factual information, previous experiences, and knowledge. Conversely, implicit, or so called procedural, memory refers to unconscious and unintentional memory, such as skilful performances, including learning to play a musical instrument or to ride a bicycle [1–3].

Many studies have suggested that distinct neural mechanisms underlie explicit and implicit memory [4–7]. The hippocampus and temporal-parietal cortex promote explicit learning and the representation of knowledge [4, 5], whereas the cortical-subcortical circuit, including the frontal cortex and basal ganglia, is attributed to implicit learning and memory [6–8]. Positron emission tomography (PET) and functional magnetic resonance imaging (fMRI) have identified a dissociation of neural substrates between implicit and explicit memory using the serial reaction time task (SRTT) [9–12]. PET results have shown that activity in the striatum is associated with the implicit condition and increased activation in the anterior cingulate, and prefrontal cortices is associated with the explicit condition [9, 10].

Yang and Li [13] found that different brain network connectivity underlies these two types of learning: explicit memory engages a network with the insula as a key mediator, whereas implicit memory directly invokes the frontal-striatal network. On the other hand, fMRI studies have reported overlapping activation in the striatal and frontal regions during both the implicit and explicit block types [11, 12], which is inconsistent with the theory of neural substrate dissociation between the two memory types. Nevertheless, most studies have shown evidence that separate neural mechanisms underlie explicit and implicit memory in the whole brain. However, few studies have sought to identify this dissociation in the hippocampus.

The hippocampus is an important structure that promotes learning and memory. It is composed of four cornu ammonis regions (CA1–4), the dentate gyrus (DG), and the subiculum (Sub). Understanding any functional specialisation within the hippocampus may be critical to further elucidate the neural basis of memory. In addition, this finding could have significant clinical implications for disorders involving hippocampal dysfunction, including dementia, Korsakov’s syndrome, and mild cognitive impairment (MCI) [14]. Accordingly, many researchers have begun to explore functional differences among individual hippocampal subregions [15–19], and Moser and Moser [16] reported that the anterior third of the hippocampus is functionally distinct from the posterior two thirds, based on animal studies. More recently, neuroimaging studies have supported that there is spatial dissociation along the anterior-posterior axis of the hippocampus during encoding and retrieval processes [15, 17–19]. Previous 3 T fMRI studies have identified that the CA2, CA3, and DG support the encoding of novel face-name and object-object associations, whereas the subicular cortices support the retrieval of these learned associations [18–20]. Zaidel et al. [21] attempted to identify the differential roles of the hippocampal subfields in the two memory types (i.e. implicit memory vs. explicit memory) by evaluating the neuronal density in hippocampal subfields correlated with each memory type and found that the left subfield CA 1 was involved in both explicit and implicit memory. However, no differences in the implicit and explicit memory processing in the hippocampal sub-regions were observed. To date, many studies have aimed to elucidate whether different regions along the hippocampal formation make a distinct contribution to memory types and stages. However, finding the distinctive roles of hippocampal sub-regions remains challenging owing to the low sensitivity of the MR signals in acquiring sufficient resolution to identify individual subfields of the hippocampus when using a 3 T MRI scanner.

Over the last 5 years, ultra-high field 7 Tesla MRI (7 T) of human brain structure and function has improved. The most prominent benefit of ultra-high magnetic field strengths in MRI is the approximately linear enhancement of the image signal-to-noise ratio (SNR) [22, 23]. In addition, BOLD-weighted functional MRI application has gained from increased susceptibility contrasts. A significant positive relationship between field strength (comparison of 1.5, 3, and 7 T) and significant voxel counts, *t* values, and amplitude of signal change has also been reported [22, 24].

This study aimed to determine which subregions in the hippocampus are involved during encoding and retrieval in implicit and explicit memory. We used ultra-high-resolution 7-T fMRI while individuals learned and recalled sequence information. We developed new tasks for this study by modifying the SRTT from Schendan and colleagues [11] to examine four types of memories, such as implicit encoding, explicit encoding, implicit retrieval, and explicit retrieval.

## Methods

### Participants

A total of 25 right-handed volunteers were recruited to participate in the present study (mean age = 24.2, SD = 3.7 years). Exclusion criteria were < 18 or > 30 years old, history of psychiatric disorders, as measured by a structured interview, such as anxiety, depression, dementia, MCI, and Korsakoff syndrome; currently using medication, history of serious head injury, and ineligible for MRI scan (i.e. has metal in the body, severe astigmatism, or claustrophobia). All participants provided written informed consent after they understood the content of the present study. The Korea Basic Science Institute Institutional Review Board (IRB) approved the experimental and consent procedure (approval number: KBSI-IRB-2017-01). All participants received financial compensation ($60) for their participation.

### Experimental task

**Fig. 1:**
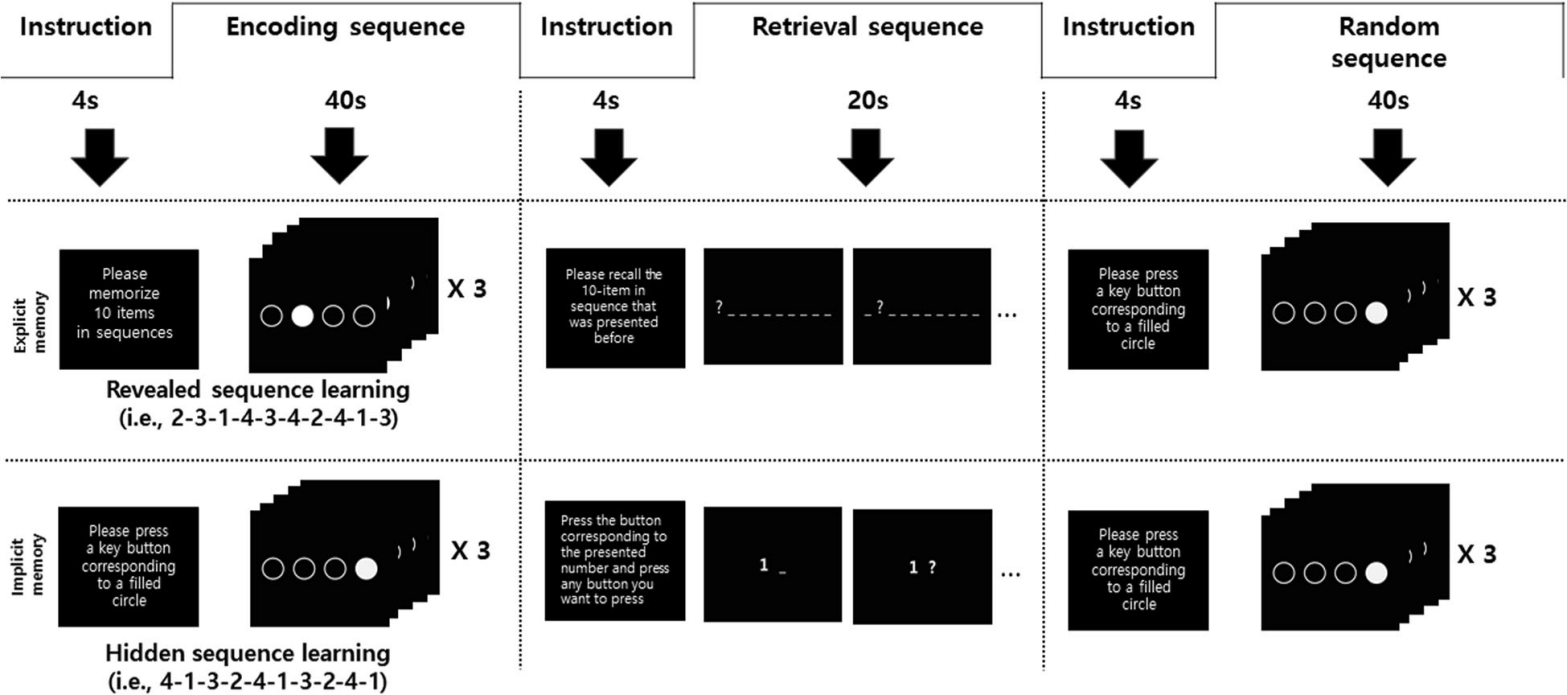
The block-designed fMRI paradigm. Explicit memory and implicit memory tasks comprise three conditions: encoding sequence (ES); retrieval sequence (RTS); and random sequence (RS). The three conditions were repeated three times for each task

Participants were asked to perform 2 block design tasks: an implicit and an explicit memory task (Fig. 1). The tasks were adapted from the SRTT, based on Schendan et al. [11]. Each task consisted of 9 blocks (3 memory encoding conditions, 3 retrieval conditions, and 3 random conditions). The sequence of stimulus presentations was as follows: (1) Participants were presented with a general description of the experiment for 4 s. (2) Instructions for the first block task (i.e. memory encoding sequence, ES) were given for 4 s. (3) Participants were given 40 s to perform the first block task. (4) Instructions for the second block task (i.e. memory retrieval sequence, RTS) were given for 4 s. (5) Participants were given 20 s to perform the second block task. (6) Instructions for the third block task (i.e. random sequence, RS) were given for 4 s. (7) Participants were given 40 s to perform the third block task. (8) Steps 2–7 were repeat 3 times. (9) Finally, instruction regarding the end of the experiment was given for 4 s. The total duration of all tasks was 340 s. In this task, the duration of the RTS block was shorter than that of the other blocks; the use of different block durations helps in eliciting optimal brain reactions according to different experimental conditions [25]. However, because the number of button presses is related to input neural firing rates, the number of buttons presses was the same in all blocks.

### Explicit memory tasks

The explicit memory task consisted of three conditions: the encoding sequence (ES), retrieval sequence (RTS), and random sequence (RS). The ES condition began with the following instruction that was presented for 4 s: “Please memorise 10 items in sequence.” Following this, four white circles were displayed. For each trial, one of the four circles changed colour to black for a duration of 1 s, followed by a blank screen for 250 ms. The four circles corresponded to keys located on the right-hand button box in which each button was to be pressed (i.e. first circle 1—button 1, second circle—button 2, third circle—button 3, and forth circle—button 4). Participants were instructed to press the corresponding button to the black circle appeared on the screen. In the ES condition, the stimuli were presented in a 10-item sequence (e.g. 2-3-1-4-3-4-2-4-1-3), which was repeated three times yielding 30 trials in total. After 10 trials, an instruction stating that “The 10-item sequence that was presented before is going to be repeated” was presented for 1450 ms. The ES condition tasks lasted 40 s. Following ES, the RTS condition began with an instruction, stating “Please recall the 10-item in the sequence that was presented before,” which was presented for 4 s. Next, a middle of the screen display of 10 hyphens was presented. Each hyphen was changed to a question mark from the first to the 10th hyphens and lasted for 2 s. When the first hyphen changed to a question mark, the participant pressed the corresponding key (i.e. button 2) introduced during the ES condition. Similarly, when the second hyphen changed to a second question mark, the participant pressed the button corresponding to that (i.e. button 3) and so on. To match the number of buttons presses in all blocks, subjects were asked to repeatedly press the recalled number three times for 2 s after the hyphen was presented.

In the RS condition, participants were instructed and given 4 s to press the button corresponding to the position of a filled circle that was presented randomly. All conditions were repeated three times.

### Implicit memory tasks

The implicit memory tasks also consisted of ES, RTS, and RS. The ES condition began with an instruction presented on the screen, stating “Please press the button corresponding to a filled circle,” for 4 s. Next, four white circles that were arranged horizontally were displayed in the middle of a screen. For each trial, one of the four circles changed colour to black for a duration of 1 s, followed by a blank screen for 250 ms. In the ES condition, the stimuli were presented in a prearranged 10-item position sequences (i.e. 4-1-3-2-4-1-3-2-4-1) that were unknown to the participants. This was repeated three times yielding to 30 trials in total. At the end of every 10 trials, an instruction was presented for 1450 ms, stating “Please press the button corresponding to a filled circle.” The ES condition tasks lasted 40 s. The RTS condition began with an instruction presented on the screen, stating “Press the button corresponding to the presented number and press any button you want to press when the hyphen next to the number turns into a question mark.” To match the number of buttons presses in all blocks, subjects were required to first press the number presented and subsequently press the recalled number twice after the hyphen was presented.

These tests were for procedural memory, which is the ability to learn an automatic sensory motor skill sub-consciously. Participants with an accuracy rate below 50% in the RTS condition were deemed not to have acquired procedural memory and were excluded from data analysis. In the RS condition, participants were instructed and given 4 s to press the button corresponding to a filled circle that was presented randomly. All conditions were repeated three times.

### Experimental procedure

Participants were provided with instructions and completed a practice session before entering the scanner to determine whether they understood the task. Once participants were settled inside the scanner, they had an opportunity to adjust their screen and become accustomed to the button presses. Following this, participants completed a practice session for each memory task prior to any fMRI data collection. Next, participants completed the explicit and implicit memory tasks during image acquisition. Each task lasted approximately 6 min with a 3-min break between tasks. For each subject, the runs of the task were presented randomly.

### Image acquisition

A 7.0 T Philips Achieva MRI scanner (Philips Medical Systems, Best, The Netherlands) was used for image acquisition. T1-weighted anatomical images were obtained using the following parameters: repetition time = 5.5 ms; echo time = 2.6 ms; flip angle = 7°; field of view = 234 × 234 mm^2^; in plane resolution = 0.7 × 0.7 × 0.7 mm^3^; 334 slices.

During the fMRI scanning, 24 continuous slices of blood-oxygen level-dependent (BOLD) images were acquired with a single-shot, echo-planar pulse sequence (repetition time = 2000 ms; echo time = 17 ms; flip angle = 70°; slice thickness = 3 mm, no gap; field of view = 192 × 198 × 72 mm^3^; in plane resolution = 1.5 × 1.5 × 3 mm^3^).

### Statistical analyses

#### Analysis of task performance

Paired *t* tests were performed to test the difference in the error rates (ERs) between explicit encoding and implicit encoding and implicit and explicit retrieval using SPSS 21.0 (IBM Corp., Armonk, NY).

#### Hippocampal segmentation

The FreeSurfer software version 6.0 (http://surfer.nmr.mgh.harvard.edu) was used for subcortical reconstruction and segmentation. Motion correction, intensity normalisation, automated topology corrections, and automatic segmentations of the cortical and subcortical regions were conducted as described previously [26, 27]. A recent automated algorithm from FreeSurfer was used to segment the hippocampal subfields. This newer version (FS 6.0) predicts a more accurate location of the hippocampal subregions using a reliable probabilistic atlas based on a combination of manual delineations of the hippocampal formation, ex vivo MRI scans, and manual annotations of the surrounding subcortical structures from in vivo, T1-weighted, 1-mm resolution MRI scans. Prior versions of the algorithm (FS5.1 to FS5.3) combined a single probabilistic atlas with high-resolution, T1-weighted in vivo manual segmentations to predict the locations of the hippocampal subregions [28]. In the current study, using segmentation, we created three hippocampal regions of interest (ROIs): CA1–3, Sub, and DG.

#### Analysis of functional imaging data

SPM12 (Wellcome Department of Imaging Neuroscience, London, UK) was used to analyse all fMRI data. Image pre-processing was performed as follows: slice-timing correction for interleaved acquisition, motion correction, and spatial normalisation onto the standard Montreal Neurological Institute template. Finally, the normalised images were smoothed with a 5-mm Gaussian kernel.

To extract the functional data from the hippocampal sub-regions, each ROI from the anatomical data of the participants was transformed to MNI coordinate by using the same transformation matrix used for the functional data. To acquire a single ROI of each anatomical region for all participants, a combined mask of the unique individual ROIs was created, preserving the maximal extent of each ROI across all participants [29].

Following completion of pre-processing, first-level statistical analysis was performed using the general linear model (GLM) to measure differences in fMRI BOLD responses between the memory (ES, RTS) and control conditions (RS) of the SRTT task for each subject. The first 8 s of each session (i.e. instruction of the task) was not included in the GLM in order to suppress the equilibration effects of the MR signal. For optimal fMRI results, motion correction parameters were added as covariates in the GLM, and the BOLD response was modeled for 40-s blocks of the ES and RS conditions and 20-s blocks of the RTS condition. For identification of areas with each memory condition-related activation, design matrices with three conditions related to implicit memory (ES, RTS, and RS) and three conditions related to explicit memory (ES, RTS, and RS) were created for each participant. The GLM contrasted activation during the memory condition (ES, RTS) was relative to the control condition (RS) in order to determine task-related fMRI activation during task processing.

Individual first-level analyses of the comparisons of the memory condition (implicit ES, implicit RTS, explicit ES, explicit RTS) and control condition (RS) were used for the random effects analysis, and mean images were created for each participant. One-sample *t* tests were performed to test for the commonality of each condition using the contrast images created in the individual analyses.

Conjunction analyses were used to identify commonalities shared across stages for each condition. For example, to locate areas activated during the encoding condition for both implicit and explicit tasks, we performed a conjunction of the results for the contrasts [implicit encoding > implicit random] and [explicit encoding > explicit random]. The same method was applied to locate a common area across the retrieval trials. Conjunction analyses were performed separately for each memory type. A combined threshold of *p* < 0.05 (FDR corrected for multiple comparisons) was considered significant in the conjunction analyses.

We measured the degree of activation using the mean % signal change extracted from each of the three ROIs for each individual. For each memory condition (i.e. implicit ES, implicit RTS, explicit ES, and explicit RTS), t-statistic map of the memory feature was calculated in a general linear model analysis at the first level. Using the ROI tool box in Marsbar 0.43 (http://marsbar.sourceforge.net), the % signal change for a specified ROI was calculated for the memory condition versus random condition. The duration for each condition was provided for this calculation. This procedure was repeated for each ROI. We performed analyses of variance (ANOVA) comparing the memory type and stage with hippocampal activation (e.g. memory type [implicit vs. explicit] and stage [encoding vs. retrieval]), within the ROIs using SPSS Version 25. We also calculated the number of voxels activated (*p* < 0.001, no correction) in each of the three hippocampal ROIs for each individual [30]. These methods allowed us to identify the regions associated with each memory condition within each hippocampal subregion.

## Results

### Results of the task performance

Behavioural data showed that 23 of 25 participants performed the encoding and retrieval tasks correctly (i.e. their performance was better than chance). During the encoding and random conditions, regardless of memory type (implicit and explicit memory), the ERs were < 10% for all participants (mean ± SD = 2.1% ± 3.4). The mean ERs of explicit and implicit retrieval were 22.7% (SD = 8.83) and 31.04% (SD = 12.77), respectively. There was no difference in ER between explicit and implicit encoding (*t*(22) = 0.19, *p* > 0.05); however, a significant difference in ER was found between explicit and implicit retrieval (*t*(22) = 2.56, *p* < 0.05). This indicated that the participants made more errors during implicit retrieval compared with explicit retrieval.

### Imaging data results

#### Hippocampal activation

**Fig. 2:**
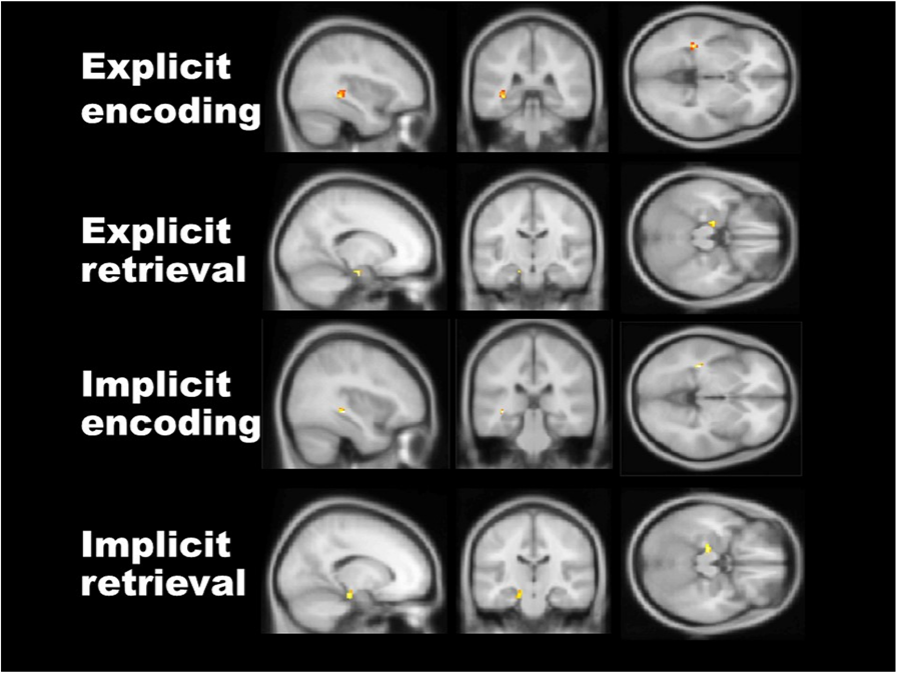
Hippocampal activation corresponding to each memory type and stage. All kinds of memories, such as explicit encoding, explicit retrieval, implicit encoding, and implicit retrieval, evoke hippocampal activation (*p* < 0.05, FDR-corrected). MNI coordinates: explicit encoding (− 38, − 18, − 14), explicit retrieval (− 18, − 14, − 22), implicit encoding (− 36, − 36, − 4), implicit retrieval (− 18, − 22, − 18)

All participants showed significant activation in the hippocampus while they performed the memory task (*p* < 0.05, FDR-corrected). Figure 2 shows the group result in hippocampal activation for the four memory types: implicit encoding, explicit encoding, implicit retrieval, and explicit retrieval (*p* < 0.05 FDR-corrected, Table 1). In all stages, activation was shown bilaterally during the explicit memory task; however, activation was observed mainly on the left side during the implicit memory task. Regardless of memory type, activation was found in the more lateral, dorsal, and posterior portions of the hippocampus during encoding when compared with retrieval. In addition, there was a distinct functional separation in the hippocampus according to the two memory types in the encoding stages. Specifically, activation induced by explicit encoding was located more anteriorly and ventrally in the hippocampus than it was in implicit encoding. Conversely, overlapping activation in the left hippocampus was observed during both implicit and explicit memory types in the retrieval stages (Table 1).

**Table 1:** Activation of each memory condition in hippocampus (*p* < 0.05, FDR-corrected)

Memory condition	Hippocampal subregions	Side	No. of voxels in cluster	*F*	x, y, z MNI coordinates		
Explicit memory task							
ES	CA 1–3	L R	59 46	3.70 3.61	− 38 34	− 18 − 24	− 14 − 16
RTS	CA 1–3	L R	45 14	3.56 3.19	− 18 16	− 14 − 20	− 22 − 18
	Subiculum	L R	16 17	3.16 3.23	− 20 20	− 22 − 18	− 18 − 18
Implicit memory task							
ES	CA 1–3	L	30	3.48	− 36	− 36	− 4
RTS	CA 1–3	L	28	3.42	− 18	− 22	− 18
	Subiculum	L	15	3.13	− 20	− 18	− 18

*ES* encoding sequence, *RTS* retrieval sequence, *R* right, *L* left

#### Common neural correlates associated with memory type and stage

**Fig. 3:**
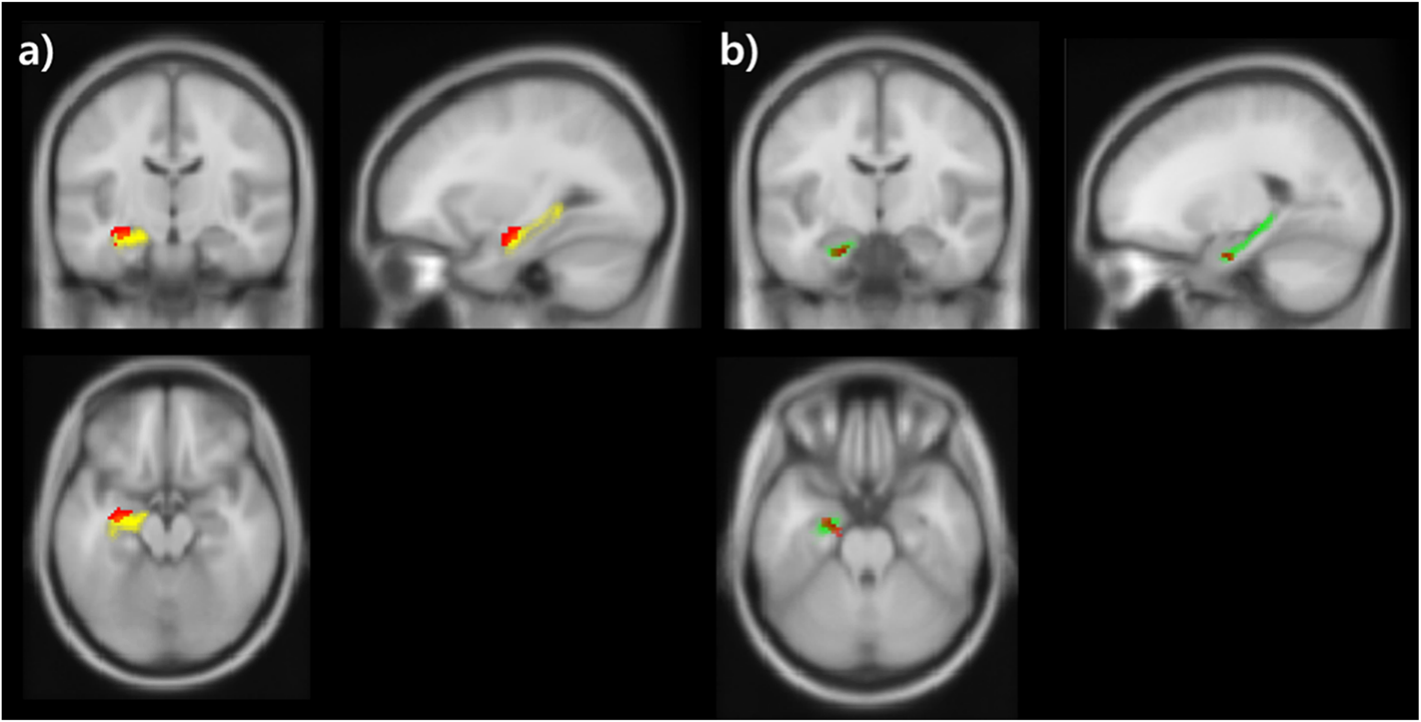
Conjunction analysis results. a Activation in the CA 1–3 is shown during encoding. b Retrieval has evoked activation of the subiculum (yellow: CA 1–3 area, green: subiculum, red: activated area). MNI coordinates: encoding (− 36, − 14, − 16), retrieval (− 18, − 10, − 24)

As shown in Fig. 3, we identified a common region that was associated with each memory type during encoding and retrieval processing using the conjunction analysis procedure (second level conjunction analysis between implicit and explicit memory). However, there was no difference in the activated region between implicit and explicit memory during all memory stages.

Next, we examined the common neural regions in which activity was associated with each memory stage during implicit and explicit memory and observed that the CA1–3 area was significantly activated during encoding when compared with retrieval. Further, we confirmed the specific involvement of the Sub in the retrieval.

#### Activation in the hippocampal subregions

**Fig. 4:**
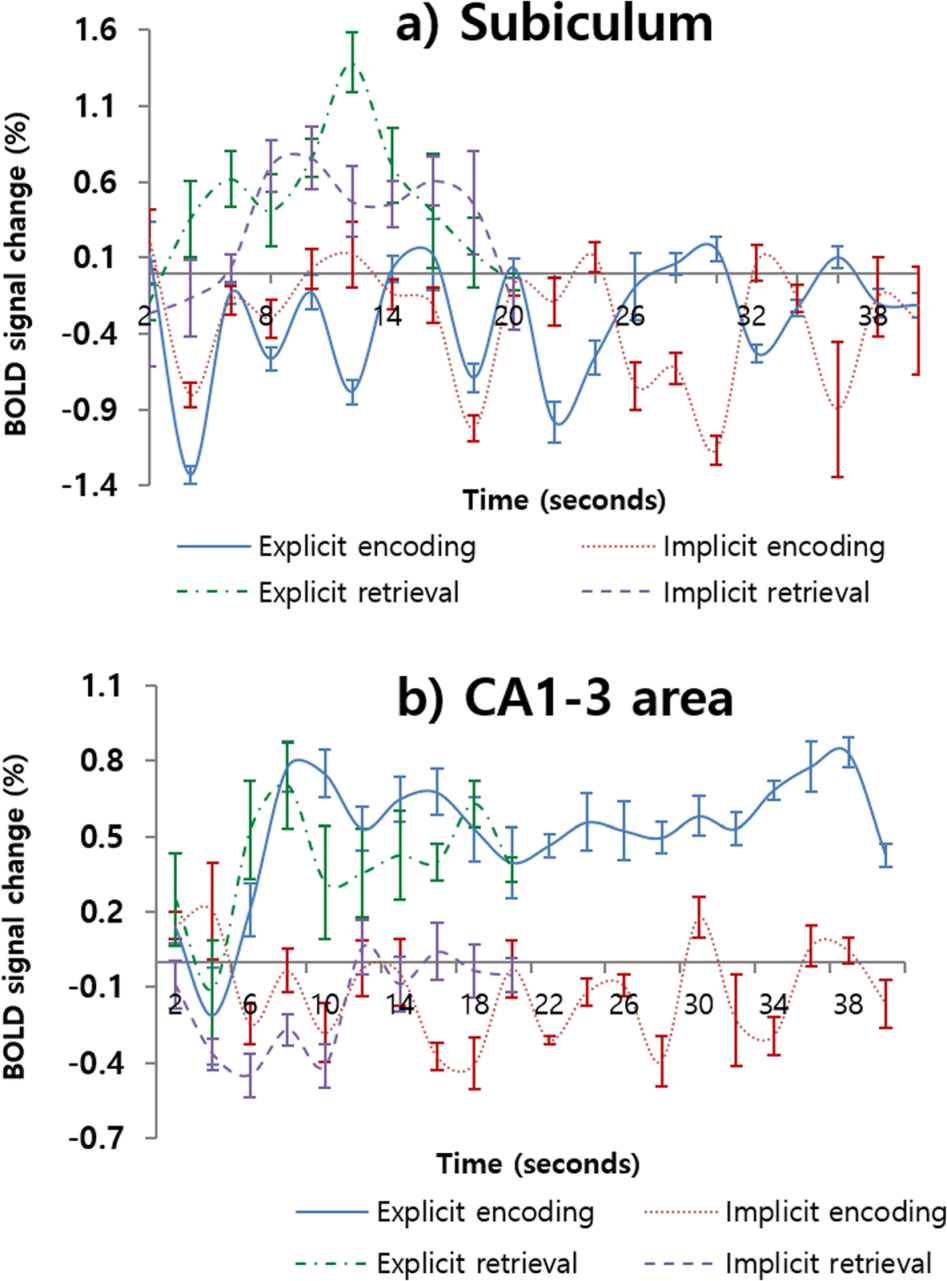
Time course of hemodynamic responses in subiculum and in the CA1–3 areas. a subiculum and b CA1–3 areas. Blue and red represent explicit encoding and implicit encoding, and green and violet indicate explicit retrieval and implicit retrieval, respectively. The y-axis and x-axis display percent signal change and number of scans each, and the error bars represents SEM

To investigate functional dissociation of hippocampal subfields, we performed a two-way ANOVA with memory type (implicit vs. explicit memory) and stage (encoding vs. retrieval) using the % signal change extracted from each hippocampal sub-region. We observed a significant primary effect of memory stage (encoding vs. retrieval) in the Sub (*F*(1, 22) = 17.32, *p* < 0.001), which showed greater activation during memory retrieval (mean = 0.37, S.D. = 0.42) than during memory encoding (mean = − 0.31, S.D. = 0.41). However, no significant primary effect of memory type (*F*(1, 22) = .39, *p* > 0.05) or a significant memory type and stage (*F*(1, 22) = .17, *p* > 0.05; Fig. 4a) interaction in the Sub was observed. Additionally, in CA 1–3, a significant effect of memory type was observed (*F*(1, 22) = 19.86, *p* < 0.001). There is greater activation during explicit memory (mean = 0.47, S.D. = 0.24) than implicit memory (mean = − 0.13, S.D. = 0.19). However, a primary effect of memory stage (*F*(1, 22) = .43, *p > 0.05*) and an interaction effect (*F*(1, 22) = .10, *p* > 0.05) were not observed (Fig. 4b). In the DG, there was no significant primary effect of group (*F*(1, 22) = 0.82; *p* > 0.05) and stage (*F*(1, 22) = 0.37; *p* > 0.05) and no interaction (*F*(1, 22) = 3.56; *p* > 0.05) on the % signal change.

**Fig. 5:**
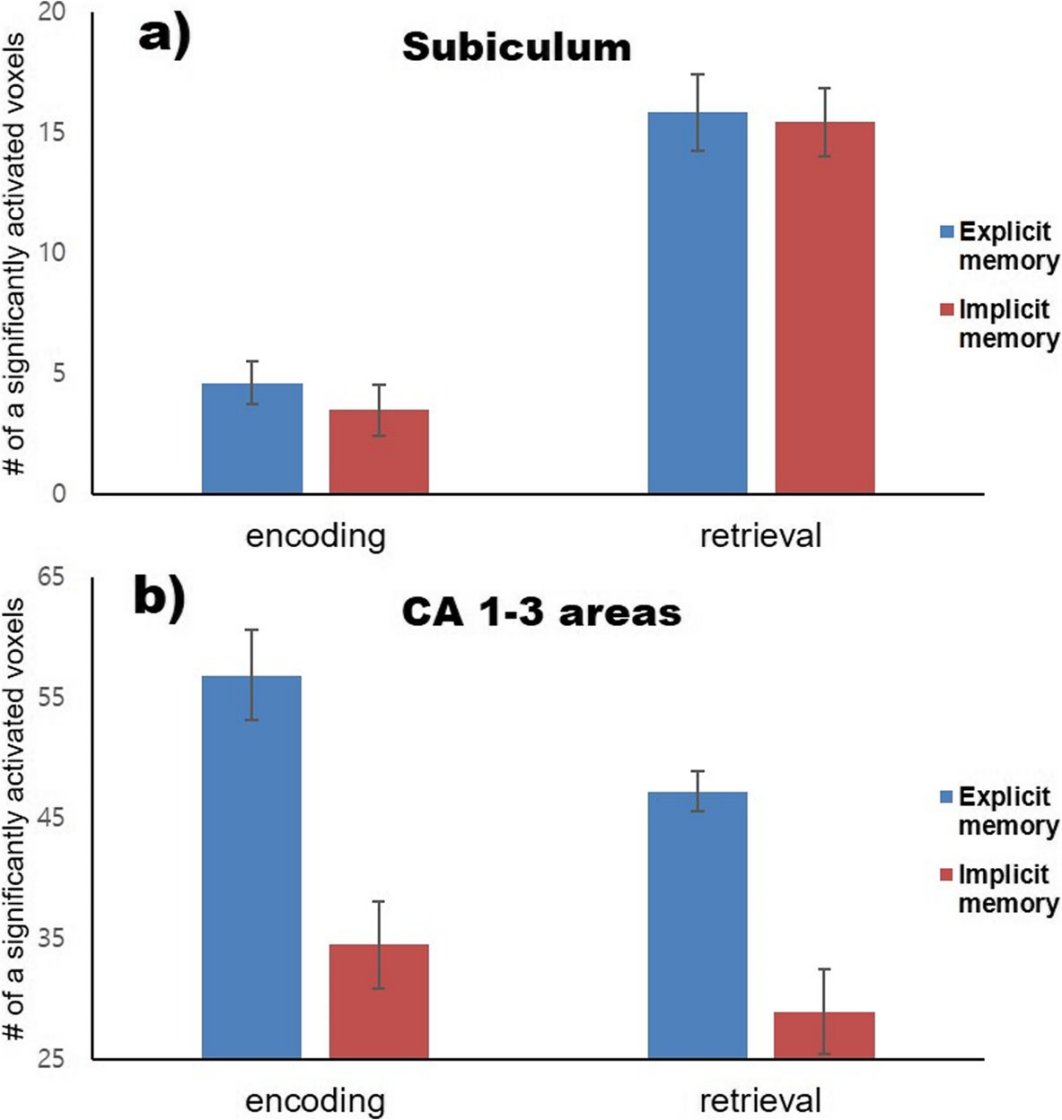
The number of significantly activated voxel in the subiculum and in the CA1–3 areas during each memory type and stage. Blue represents explicit memory, and red indicates implicit memory. a In the subiculum, the main effect of memory stage on the number of significantly activated voxel was found. b There are significant main effects of memory type and stage in the CA1–3 areas

Additionally, we conducted a two-way ANOVA using the number of activated voxels. In the Sub, there was a significant main effect of memory stage on the number of significantly activated voxels (*F*(1, 22) = 82.59, *p* < 0.001); however, there was no main effect of memory type (*F*(1, 22) = 0.36; *p* > 0.05) or interaction (*F*(1, 22) = 3.14; *p* > 0.05; Fig. 5a). In CA1–3, there was a significant main effect of memory type (*F*(1, 22) = 39.13, *p* < 0.001) and stage (*F*(1, 22) = 5.51, *p* < 0.05) on the number of significantly activated voxels. However, there was no significant interaction (*F*(1, 22) = 0.39; *p* > 0.05; Fig. 5b). In the DG, there was a nonsignificant main effect of group (*F*(1, 22) = 0.7; *p* > 0.05) and stage (*F*(1, 22) = 0.51; *p* > 0.05) and no interaction (*F*(1, 22) = 1.28; *p* > 0.05) on the number of significantly activated voxels.

## Discussion

In this study, we examined the functional dissociation of hippocampal subregions corresponding to memory types (i.e. implicit and explicit memory) and stages (i.e. encoding and retrieval) using ultra high-resolution 7-T fMRI. First, we found activation in the hippocampus associated with all memory types and stages. Interestingly, we found that distinct hippocampal regions were activated according to each memory type; activation during encoding was found in more lateral, dorsal, and posterior regions of the hippocampus compared with retrieval regardless of memory type. Second, we found differences in activation between memory types at each memory stage. During explicit encoding, hippocampal activation was shown more anteriorly and ventrally when compared with implicit encoding. Conversely, overlapping activation in the left hippocampus was found in both implicit and explicit memory tasks in the retrieval stage. Third, we identified the hippocampal sub-regions corresponding to different memory stage and type via conjunction analysis. Using this analysis, although we are unable to locate any specific region corresponding to implicit and explicit memory, we confirmed the specific involvement of the Sub in the retrieval process. Fourth, we examined the amount of activation based on the BOLD signal change and the number of voxels to identify specificity in the hippocampal subareas for each memory type and stage. We found that the Sub was associated with retrieval, while the CA1–3 was linked to explicit memory.

Currently, there is some debate regarding the involvement of the hippocampus in implicit memory. Previous studies have reported that the hippocampus is associated with conscious/explicit long-term memory for previously experienced events [31–36]. Participants with lesions in the hippocampus and adjacent medial temporal lobe (MTL) showed poor performance on explicit/direct tests of long-term memory (i.e. recognition and recall) when compared with implicit/indirect tests, such as priming [37–40]. This has also been demonstrated in various fMRI studies; explicit, but not implicit, memory consistently activates the hippocampus [33, 34]. Other neuroimaging studies testing recognition memory for complex visual scenes have reported activity in the MTL, including the hippocampus, only during explicit memory tasks. These results support the influential view that the MTL, including the hippocampus, plays a key role in explicit memory [31, 32, 35, 36].

In contrast, some studies have suggested that scene learning and discrimination are selectively disrupted in hippocampal amnesia during implicit/indirect tasks [41, 42]. Additionally, some fMRI studies have reported that there is a hippocampal contribution during implicit memory [11, 43, 44]. This activation was shown using the face-scene association, visual detection, and SRTTs; therefore, indicating that unconscious processing of memory tasks evokes hippocampal activity. In line with these studies, we identified hippocampal activation during implicit and explicit memory processing, regardless of memory stage using a SRTT. Taken together, this indicated that the hippocampus is critical for the unconscious formation and retrieval of relational information.

Moser and Moser [16] suggested that the hippocampal structure is functionally heterogeneous, with different portions of the longitudinal axis promoting discrete functional roles, because of differences in connectivity. Consistent with this, we showed a functional dissociation in hippocampal subregions according to memory type and stage. Activation of the CA 1–3 was associated with both creation of a memory representation and retrieval of memories, whereas the Sub was engaged during retrieval but not during the encoding of memory. These results are consistent with previous results that report anatomically unspecified activity in the CA1–3 region during both encoding and retrieval [20, 45–47]. In line with our results, Eldridge and colleagues [20] suggested that activation in the CA2 and 3 regions during encoding is associated with both recollection and familiarity-based recognition, whereas the Sub was active during the recollection of the learning episode. Also, in a previous study using spatial memory task, the Sub activity increased during retrieval compared to encoding of spatial association [48]. Our results are also supported by previous neuroimaging studies using block designed paradigms, which observed Sub activities only during retrieval [19, 20, 31, 48].

Functional dissociation among hippocampal subregions is supported by molecular and cellular biology studies. Roy et al. [49] created a mouse line expressing Cre recombinase under the promoter for the Fibronectin 1 gene to distinguish different memory functions of the CA1 and Sub, which were previously reported to be expressed almost entirely in the dorsal Sub. Monosynaptic rabies virus tracing and histological studies have confirmed that Cre positive cells are innervated by generally known inputs to the dorsal Sub, and they express markers for excitatory, and not inhibitory, neurons. In that study, Roy et al. [49] suggested that CA 1 and the Sub were implicated in complementary roles, with the direct CA1-cortical projection supporting memory encoding and the subicular–cortical projection being more involved in retrieval. In sum, these results including our result indicate that the Sub may be specific for memory retrieval.

There are some limitations in this study. Firstly, we found differences among memory types and stages only in the hippocampal area. According to previous studies, both in the hippocampus and in other areas including the occipital area, prefrontal cortex, and precuneus, there are marked differences in the activating pattern and brain areas between implicit and explicit memory [50–52]. However, because of the constraint of 7 T MRI, it is not possible to scan the whole brain to acquire high-resolution imaging data. In a future study, we plan to develop suitable imaging parameters and identify differences according to memory types and stages at the whole-brain level. Second, we used relatively large hippocampal subregions as ROIs. Due to the advantage of using 7 T MRI, which allows for high-resolution imaging, we initially intended to divide the hippocampus into smaller parts (i.e. CA1, CA2-3, CA4, fimbria, parasubiculum, presubiculum, subiculum, dentate gyrus, hippocampal-amygdaloid transition area, and tail). In order to distinguish the CA 1–3 areas, a resolution of at least 1.5 mm^3^ or less is required. However, regarding our epi data, image distortion was severe when the spatial resolution reached below 1.5 mm^3^, regardless of which protocol or shim tool was applied. Due to the limitations of our 7 T MRI, the optimal resolution of our epi data was 1.5 × 1.5 × 3 mm. In a future study, to acquire higher resolution functional imaging data, we plan to apply a newly developed shim tool and obtain suitable imaging. Thirdly, our study does not exclude the possibility that memory processing using visuospatial material preferentially activates the hippocampus. Baddeley and Hitch [53] suggested that there are two different systems (i.e. the phonological loop and visuospatial sketch pad) in working memory. Hence, we need to identify whether the same results can be derived from a verbal memory task. Finally, in future studies using 7 T MRI, we should consider some estimate of the susceptibility artefact. Recent work by Devlin et al. [54] and Cordes et al. [55] demonstrated that the susceptibility artefact is not limited to the temporal pole or lateral temporal regions, and it can significantly influence regional analyses of hippocampal function.

Although there are some limitations, this is the first study to identify the functional dissociation among hippocampal subregions for memory types and stages (i.e. explicit encoding, explicit retrieval, implicit encoding, and implicit retrieval) using 7 T MRI scanning. This result broadens our understanding of hippocampal function as an important hub in the memory processing and pathogenesis of psychiatric disorders including of Alzheimer’s disease and MCI.

## Conclusion

We identified that the hippocampus subserves both implicit and explicit memory processing. Moreover, we confirmed the functional dissociation between CA 1–3 and the Sub. We found that the CA 1–3 areas were implicated in both memory encoding and retrieval, whereas the Sub was only engaged in memory encoding.

## Data Availability

The data are not publicly available due to their containing information that could compromise the privacy of research participants. Requests to access these datasets should be directed to the corresponding author.

## Abbreviations

7 T: 7 Tesla MRI
BOLD: Blood-oxygen level-dependent
CA: Cornu ammonis regions
DG: Dentate gyrus
ERs: Error rates
ES: Encoding sequence
fMRI: Functional magnetic resonance imaging
GLM: General linear model
IRB: Institutional Review Board
MCI: Mild cognitive impairment
MTL: Medial temporal lobe
PET: Positron emission tomography
ROIs: Regions of interest
RS: Random sequence
RTS: Retrieval sequence
SRTT: Serial reaction time task
Sub: Subiculum

## References

[1] Parkin AJ, Reid TK, Russo R (1990). On the differential nature of implicit and explicit memory. Mem Cogn.

[2] Schacter DL (1987). Implicit memory: History and current status. J Exp Psychol Learn Mem Cogn.

[3] Ullman MT (2004). Contributions of memory circuits to language: the declarative/procedural model. Cognition..

[4] Cohen NJ, Eichenbaum H, Deacedo BS, Corkin S (1985). Different memory systems underlying acquisition of procedural and declarative knowledge a. Ann N Y Acad Sci.

[5] Eichenbaum H (1999). Conscious awareness, memory and the hippocampus. Nat Neurosci.

[6] Heindel WC, Salmon D, Shults CW, Walicke P, Butters N (1989). Neuropsychological evidence for multiple implicit memory systems: a comparison of Alzheimer’s, Huntington’s, and Parkinson’s disease patients. J Neurosci.

[7] Knowlton BJ, Squire LR, Schacter D (2002). The role of the basal ganglia in learning and memory. The Neuropsychology of Memory.

[8] Reber PJ, Squire LR (1994). Parallel brain systems for learning with and without awareness. Learn Mem.

[9] Destrebecqz A, Peigneux P, Laureys S, Degueldre C, Del Fiore G, Aerts J (2005). The neural correlates of implicit and explicit sequence learning: interacting networks revealed by the process dissociation procedure. Learn Mem.

[10] Rauch SL, Savage CR, Brown HD, Curran T, Alpert NM, Kendrick A (1995). A PET investigation of implicit and explicit sequence learning. Hum Brain Mapp.

[11] Schendan HE, Searl MM, Melrose RJ, Stern CE (2003). An FMRI study of the role of the medial temporal lobe in implicit and explicit sequence learning. Neuron.

[12] Willingham DB, Salidis J, Gabrieli JD (2002). Direct comparison of neural systems mediating conscious and unconscious skill learning. J Neurophysiol.

[13] Yang J, Li P (2012). Brain networks of explicit and implicit learning. PLoS One.

[14] Small BJ, Fratiglioni L, Viitanen M, Winblad B, Bäckman L (2000). The course of cognitive impairment in preclinical Alzheimer disease: three-and 6-year follow-up of a population-based sample. Arch Neurol.

[15] Lepage M, Habib R, Tulving E (1998). Hippocampal PET activations of memory encoding and retrieval: the HIPER model. Hippocampus..

[16] Moser MB, Moser EI (1998). Functional differentiation in the hippocampus. Hippocampus..

[17] Schacter DL, Wagner AD (1999). Medial temporal lobe activations in fMRI and PET studies of episodic encoding and retrieval. Hippocampus..

[18] Zeineh MM, Engel SA, Thompson PM, Bookheimer SY (2001). Unfolding the human hippocampus with high resolution structural and functional MRI. Anat Rec.

[19] Zeineh MM, Engel SA, Thompson PM, Bookheimer SY (2003). Dynamics of the hippocampus during encoding and retrieval of face-name pairs. Science..

[20] Eldridge LL, Engel SA, Zeineh MM, Bookheimer SY, Knowlton BJ (2005). A dissociation of encoding and retrieval processes in the human hippocampus. J Neurosci.

[21] Zaidel DW, Esiri MM, Beardsworth ED (1998). Observations on the relationship between verbal explicit and implicit memory and density of neurons in the hippocampus. Neuropsychologia..

[22] Sladky R, Baldinger P, Kranz GS, Tröstl J, Höflich A, Lanzenberger R (2013). High-resolution functional MRI of the human amygdala at 7 T. Eur J Radiol.

[23] Triantafyllou C, Hoge RD, Krueger G, Wiggins CJ, Potthast A, Wiggins GC (2005). Comparison of physiological noise at 1.5 T, 3 T and 7 T and optimization of fMRI acquisition parameters. Neuroimage..

[24] van der Zwaag W, Francis S, Head K, Peters A, Gowland P, Morris P (2009). fMRI at 1.5, 3 and 7 T: characterising BOLD signal changes. Neuroimage..

[25] Soloff, PH, White R, Omari A, Ramaseshan K, Diwadkar VA. Affective context interferes with brain responses during cognitive processing in borderline personality disorder: fMRI evidence. Psychiatry Res. Neuroimaging. 2015;233:23–35. 10.1016/j.pscychresns.2015.04.006 PMC4465042 25982488

[26] Dale AM, Fischl B, Sereno MI (1999). Cortical surface-based analysis: I. Segmentation and surface reconstruction. Neuroimage.

[27] Fischl B, Salat DH, Busa E, Albert M, Dieterich M, Haselgrove C (2002). Whole brain segmentation: automated labeling of neuroanatomical structures in the human brain. Neuron..

[28] Iglesias JE, Augustinack JC, Nguyen K, Player CM, Player A, Wright M (2015). Alzheimer’s disease neuroimaging initiative “A computational atlas of the hippocampal formation using ex vivo ultra-high resolution MRI: application to adaptive segmentation of in vivo MRI”. Neuroimage..

[29] Dickerson BC, Miller SL, Greve DN, Dale AM, Albert MS, Schacter DL (2007). Prefrontal-hippocampal-fusiform activity during encoding predicts intraindividual differences in free recall ability: an event-related functional-anatomic MRI study. Hippocampus..

[30] Greicius MD, Krasnow B, BoyettAnderson JM, Eliez S, Schatzberg AF, Reiss AL (2003). Regional analysis of hippocampal activation during memory encoding and retrieval: fMRI study. Hippocampus..

[31] Gabrieli JD, Fleischman DA, Keane MM, Reminger SL, Morrell F (1995). Double dissociation between memory systems underlying explicit and implicit memory in the human brain. Psychol Sci.

[32] Henson RNA, Cansino S, Herron JE, Robb WGK, Rugg MD (2003). A familiarity signal in human anterior medial temporal cortex?. Hippocampus..

[33] Henson R (2005). A mini-review of fMRI studies of human medial temporal lobe activity associated with recognition memory. Q J Exp Psychol [B].

[34] Montaldi D, Spencer TJ, Roberts N, Mayes AR (2006). The neural system that mediates familiarity memory. Hippocampus..

[35] Squire LR, Zola-Morgan S (1991). The medial temporal lobe memory system. Science..

[36] Squire LR, Stark CE, Clark RE (2004). The medial temporal lobe. Annu Rev Neurosci.

[37] Cohen NJ, Squire LR (1980). Preserved learning and retention of pattern-analyzing skill in amnesia: dissociation of knowing how and knowing that. Science..

[38] Milner B (1970). Memory and the medial temporal regions of the brain. Biol Memory.

[39] Penfield W, Milner B (1958). Memory deficit produced by bilateral lesions in the hippocampal zone. AMA Arch Neurol Psychiatry.

[40] Shimamura AP, Squire LR (1987). A neuropsychological study of fact memory and source amnesia. J Exp Psychol Learn Mem Cogn.

[41] Graham KS, Scahill VL, Hornberger M, Barense MD, Lee AC, Bussey TJ (2006). Abnormal categorization and perceptual learning in patients with hippocampal damage. J Neurosci.

[42] Mundy ME, Downing PE, Dwyer DM, Honey RC, Graham KS (2013). A critical role for the hippocampus and perirhinal cortex in perceptual learning of scenes and faces: complementary findings from amnesia and fMRI. J Neurosci.

[43] Degonda N, Mondadori CR, Bosshardt S, Schmidt CF, Boesiger P, Nitsch RM (2005). Implicit associative learning engages the hippocampus and interacts with explicit associative learning. Neuron.

[44] Hannula DE, Ranganath C (2008). Medial temporal lobe activity predicts successful relational memory binding. J Neurosci.

[45] Davachi L, Wagner AD (2002). Hippocampal contributions to episodic encoding: insights from relational and item-based learning. J Neurophysiol.

[46] Otten LJ, Henson RN, Rugg MD (2001). Depth of processing effects on neural correlates of memory encoding: relationship between findings from across-and within-task comparisons. Brain..

[47] Strange BA, Otten LJ, Josephs O, Rugg MD, Dolan RJ (2002). Dissociable human perirhinal, hippocampal, and parahippocampal roles during verbal encoding. J Neurosci.

[48] Suthana N, Ekstrom A, Moshirvaziri S, Knowlton B, Bookheimer S (2011). Dissociations within human hippocampal subregions during encoding and retrieval of spatial information. Hippocampus..

[49] Roy DS, Kitamura T, Okuyama T, Ogawa SK, Sun C, Obata Y (2017). Distinct neural circuits for the formation and retrieval of episodic memories. Cell..

[50] Gureckis TM, James TW, Nosofsky RM (2011). Re-evaluating dissociations between implicit and explicit category learning: an event-related fMRI study. J Cogn Neurosci.

[51] Reber PJ, Gitelman DR, Parrish TB, Mesulam MM (2003). Dissociating explicit and implicit category knowledge with fMRI. J Cogn Neurosci.

[52] Seger CA, Prabhakaran V, Poldrack RA, Gabrieli JD (2000). Neural activity differs between explicit and implicit learning of artificial grammar strings: an fMRI study. Psychobiology..

[53] Baddeley AD, Hitch G (1974). Working memory. Psychol Learn Motiv.

[54] Devlin JT, Russell RP, Davis MH, Price CJ, Wilson J, Moss HE (2000). Susceptibility-induced loss of signal: comparing PET and fMRI on a semantic task. Neuroimage..

[55] Cordes D, Turski PA, Sorenson JA (2000). Compensation of susceptibility-induced signal loss in echo-planar imaging for functional applications. Magn..

