# Correction to: Functional dissociation of hippocampal subregions corresponding to memory types and stages

**DOI:** 10.1186/s40101-020-00243-9

**Published:** 2020-10-14

**Authors:** Ji-Woo Seok, Chaejoon Cheong

**Affiliations:** 1 Department of Psychiatry, University of Nebraska Medical Center, Omaha, NE USA; 2 Department of Rehabilitation Counseling Psychology, Seoul Hanyoung University, Seoul, Republic of Korea; 3 Center for Research Equipment, Korea Basic Science Institute, 162 Yeongudanji-ro, Ochang, Cheongju, 28119 Chungbook Republic of Korea

**Correction to: J Physiol Anthropol 39, 15 (2020)**

**https://doi.org/10.1186/s40101-020-00225-x**

It was highlighted the in the original article [1] there was an error in the last sentence of the Conclusion section. This Correction article shows the incorrect and correct sentence.

**Incorrect sentence**

We found that the CA 1–3 areas were implicated in both memory encoding and retrieval, whereas the Sub was only engaged in memory encoding.

**Correct sentence**

We found that the CA 1–3 areas were implicated in both memory encoding and retrieval, whereas the Sub was only engaged in memory retrieval.
